# Animal experimentation in transgenesis: evaluating course design in large classrooms

**DOI:** 10.1002/2211-5463.12846

**Published:** 2020-04-28

**Authors:** Jean‐François Bodart, Aurélie Dupré

**Affiliations:** ^1^ CNRS UMR 8576 – UGSF – Unité de Glycobiologie Structurale et Fonctionnelle Univ. Lille France; ^2^ CIREL‐Théodile EA 4354 DIP – Service Conseil et Accompagnement à la Pédagogie Univ. Lille France

**Keywords:** animal experimentation, animal welfare, education, survey, transgenesis, value

## Abstract

Teachers are guided by an ethical code of conduct. Teacher behavior can be perceived as normative and can set standards; for example, in the field of animal experimentation. The importance of ethical standards raises the question of its transmission. This survey addressed the relevance of using large amphitheater teaching groups to educate students on the ethical aspects of animal experimentation. A course was built to include interactivity sequences to gather feedback from students about moral dilemmas or assertions about animal experimentation. To that end, surveys were conducted on third‐year students, prior to the course, shortly after the course and at the end of the academic year. Students were asked to indicate whether the experimental protocols were satisfactory. Before the course, few students reported ethical dimensions in the proposed protocols; animals were considered scientific objects, not sentient beings. The situation was noticeably different for students on courses with an emphasis on the animal as the unit of study. Although large classrooms are not considered to be relevant places to question ethical issues, the proportion of students discussing ethical aspects of protocols increased shortly after the lecture, and this increased at the end of the academic year. These observations suggest that the effect of teaching on ethical considerations was sustainable despite the lectures being performed in a large classroom.

## Graphical abstract

We addressed the relevance of using large amphitheater teaching groups to educate students in biology on the ethical aspects of animal experimentation. A course was built to include interactivity sequences aiming to gather feedback from students about moral dilemmas or assertions about animal experimentation. Through our survey, we observed that the effect of teaching was sustainable.

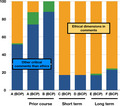

**Abbreviations**
3R: Reduction, Replacement and Refinement
BCP: Cellular Biology and Physiology
BOP: Biology of Organisms and Populations
CAPE: Teaching Practices Support Center
CITES: Convention on International Trade in Endangered Species of Wild Fauna and Flora
EAE: experimental autoimmune encephalomyelitis
HIS: Hybridization *in situ*

Lectures and courses include implicit and explicit content [1], where implicit refers to implied and unintentional content, and explicit refers to content that is clearly stated and intentional. Values might be regarded as a ‘conception of the desirable, which influences the selection from available modes, means and ends of actions’ [2]. Building upon this definition, Rokeach [3] proposed values to be abstract ideals representing a person’s beliefs about modes of conduct and ideal terminal modes, which transcendentally guide actions and judgments across specific objects and situations. The implicit values expressed in courses may freeze representations, with the risk that these values might be adopted or anchored without being formally addressed. In the sciences, knowledge is presented and perceived as a body of objective results [4]. Although an individuals' core values are set when they become adults and remain relatively stable thereafter [3], these values might be re‐evaluated in situations that create conflicts between beliefs and scientific knowledge or observations.

In the fields of science and particularly in biology, educators deal with a double challenge; they face the ethical issues in their professional lives and research activities and they aim to instill a critical spirit among students [5]. Teachers may therefore provoke situations where students (a) are invited to detect the underlying values associated with their education or culture and (b) have an opportunity to think over the involvement of these values within the considered knowledge [1]. Therefore, ethical issues have to be considered at the core of teaching activities, particularly in studies where (a) animals have been killed and dissected to demonstrate anatomical principles or (b) where living animals or organs withdrawn from them are subjected to invasive experiments (e.g. in biochemistry, cellular and molecular biology, parasitology, physiology, pharmacology). Such studies are bound to raise not only controversy but also objections, as reported in veterinary and life and health courses [6].

Animal experimentation involves making ethical decisions and is more and more widely considered as a social issue. In 1959, Russell and Burch urged researchers to promote both the quality of research and the ethical treatment of animal in their care by defining a 3R principle (Reduction, Replacement and Refinement [7]). Such responsibility, which may be considered as a fourth R [8], is shared by all those participating in the use of animals for research or for pedagogical purposes, including animal caretakers, veterinarians, inspectors, funding agencies, scientific journal editors, ethics committees, researchers, lecturers and teachers. Another potential fourth R has been proposed: Relevance [9]. The 3Rs ethical principle has played a pivotal role in researchers’ awareness and promotion of stringent regulations concerning animal use. Indeed, the wide acceptance of 3Rs has provided a roadmap for addressing most of the issues in laboratory animal welfare, and the 3Rs principle of replacement, refinement and reduction has been endorsed by legislators (EU Directive 2010/63EU) [10]. Nevertheless, the values that underline the 3Rs still need to be better stated and understood, rather than being adopted without being addressed [11].

At the pedagogical level, large classrooms are not perceived as ideal places to question ethical issues because the amount and intensity of the interactions between teachers and students is reduced as the size of the classroom increases. This can result in anonymity and passivity among students [12, 13]. In this situation, the attitude and the expertise of the teacher [14, 15] are central to the effectiveness of lectures. Lectures must be effective in a large range of contexts, including, for example, (a) when it is necessary to arouse interest into a topic; (b) when the information is original or must be integrated from different sources; (c) when the teacher wants to provide supplementary explanations of material that students may have difficulty learning on their own; and (d) when the teacher wants to include problem solving and critical thinking, relating courses to student’s personal experiences [16, 17, 18]. To counteract poor engagement, low motivation and low participation levels of the students, which are often observed in the context of large classes [19], learning environments that are centered on students can be built. Many suggestions and recommendations have been made to achieve this, including brainstorming, brief demonstrations, quick surveys, short essay writing, peer teaching, drama, debate, simulation, role playing, short presentations by students or multiple brief pauses for the students to consolidate their notes [20].

Using more active teaching in large classes remains challenging when addressing topics that are at the cross road of ethical issues. In genetics, transgenesis is such a topic because it is defined as a process of transferring genes (segment of DNA) from one organism into another cell or organism. Transgenic animal production raises not only ethical issues about animal experimentation, but also additional ethical concerns when it involves the manipulation of an embryo [21, 22], changing the human genome for therapeutic purposes [23, 24] or creating a human‐animal chimera [25].

In the present study, we report the results of a survey performed among third‐year undergraduates majoring in Physiology and Cell Biology. Our goals were (a) to promote awareness of animal welfare through the understanding of the scientific method; (b) to evaluate students’ attitudes in large classrooms towards ethical issues; (c) to assess the impact of interactivity in large classes on students' attitudes and critical thinking towards experimental protocols; and (d) to assess the desire of students to exercise critical thought on experimental protocols. The results obtained were compared with those obtained with third‐year bachelor students majoring in Biology of Organisms and Populations (BOP), who were taught in a different manner and had a different background and approach towards animal experimentation.

## Materials and methods

### Participants

The course entitled ‘Animal Transgenesis’ is mandatory for all third‐year undergraduate students majoring in Cellular Biology and Physiology (BCP). In 2016, students were enrolled in a survey. Comments of the students to the protocols were collected and several types of comments were observed: reminders of legislation regarding animal experimentation or protected species, discussion of the ethical nature of the protocol (3R), an empathic response (consideration of animal suffering) and even the refusal of any animal experimentation.

Students were surveyed using a printed questionnaire. At the beginning of each course where a survey is carried out, a speaker, who was not known to the students, introduced himself or herself. He or She explained that they were a member of the Teaching Practices Support Center (CAPE) and asked the students if they would agree to answer to a survey. Participation was voluntary and was not linked to any particular teaching module. The survey was manually distributed in the amphitheater, in paper format, to each student, who was free to refuse. Students had 20 min to read and write their comments to the proposed protocols. The students who agreed to receive and return the survey were counted (even if there was no response to the survey).

The survey involved up to 137 third‐year undergraduates majoring in BCP (2016). Eighty‐nine third‐year undergraduate who majored in BOP (2016) and did not take a course on animal transgenesis were also enrolled in the survey.

### Ethics

No identifiable or confidential information was collected. No gender, age or other demographic factors were requested or considered within the analysis. Participation in the study was optional, as stated in the section ‘participants’. Students were informed that the survey was being performed for the CAPE, and that it was unrelated to the courses and was not connected to their grades.

### Procedures for surveys

To estimate the effectiveness of the different teaching approaches towards the displayed objective, we assessed students' sensitivity to ethics issues at three points in the academic year: (a) in October 2016, when the lecture course concerning ethics issues began; (b) at the end of the lectures on transgenesis (December 2016); and (c) at the end of Spring semester (April 2017), 8 months after the lecture on transgenesis. These evaluations were carried out during lectures in the amphitheater. To ensure that these evaluations are not associated with ethics teachings, the surveys were not passed out in courses provided by the teachers involved in the lecture on transgenesis. The cohort of students majoring in BOP was surveyed in December 2016.

Surveys were collected anonymously to promote honest responses.

To assess sensitivity to ethics issues, we asked students to respond, at each time point during the academic year, to two experimental protocols with potential ethical problems. The students were asked at the end of each protocol if the experimental protocol presented was satisfactory (see ‘Protocol Texts for Surveys’ below). Students were asked to justify their response. We deliberately chose not to explain the ethical problem in the question to determine whether ethics appeared spontaneously in their comments and remarks.

The results of the survey were analyzed by a single person to avoid any source of variability in interpretation. The responses including either only the word ethics, without any supportive statements, or statements about animal suffering, legislative knowledge, or one or more of the 3Rs, were grouped in a category ‘students mentioning an ethical dimension’. The group ‘no argument’ corresponds to students who wrote a comment, but did not develop it, such as ‘the protocol is not ethical’. Answers of type ‘yes’, ‘no’ and ‘I don't know’, which did not develop an argument, were placed in the category ‘no discussion’. The answers of the students who developed an argument other than ethical were placed in a third category.

### Course and vote

**Fig. 1:**
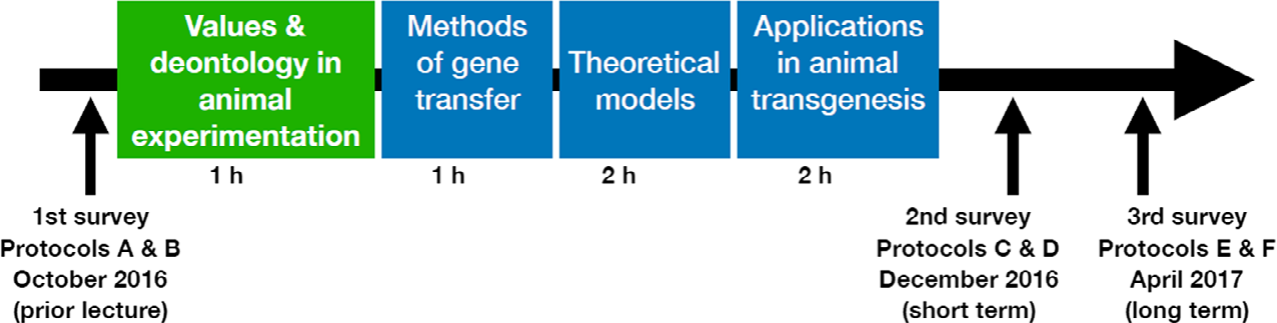
Timescale of the lectures and surveys. Lecture details are provided in Table 1.

The lecture on transgenesis was given to third‐year undergraduate students majoring in BCP. The course timetable and milestones of the lecture are provided in Table 1 and the timescale of lecture and surveys is shown in Fig. 1. Several premises questioning values were proposed to the students and subjected to a vote. The results of the votes were collected during the courses in 2017, 2018 and 2019. This scheme included interactivity by polls within large groups, as well as changes in the educator position. Votes were performed using the platform http://toreply.univ-lille1.fr, which enables anonymous responses.

**Table 1:** Course timetable and milestones.

Course timetable and milestones				
Introduction	Definition of transgenesis; from correlation to causality in animal models: towards experimental genetic; Animal models are analogical one			10 min
Implicit values in animal experimentation and valuation exercise (Part 1 – Interactive Sequence)	1st proposal	‘It’s wrong to kill one of our own’	Vote: true/false	25 min
	1st moral dilemma	At the helm: ‘you are the captain of a ship in the open sea carrying a thousand sailors on board. A fire broke out in the engine room and the only way to extinguish this fire is to cut off the oxygen in this room. This oxygen cut will result in the death of four sailors in this room. As captain, do you have to turn off the oxygen? ’. Two options are proposed: (a) You have to turn off the oxygen (b) you must not turn off oxygen	Vote (a) I have to turn off the oxygen (b) I must not turn off oxygen	
	2nd moral dilemma	Trolley problem: ‘You are witnessing a runaway trolley moving toward five tied‐up people lying on the tracks. You are standing next to a lever, which controls a switch. If you pull the lever, the trolley will be redirected onto a side track, and the five people on the main track will be saved. However, there is a single person lying on the side track.’ Two options are proposed: (a) you do nothing and allow the trolley to kill the five people on the main track or (b) you pull the lever, leading the trolley onto the side track where it will kill the one incapacitated person on the rails	Discussion phase	
	2nd proposal	Animals are one of us	Vote: true/false	
	3rd proposal	Embryos are one of us	Vote: true/false	
Deontology (Part 2 – Milestones & Laws)	Descartes (1637); Bentham (1834); Singer (1975); Nussbaum (2004); Donaldson & Kymlicka (2016) 3 R Rules (Russell and Burch, 1959) Ethical recommendations and laws applied in France Alternative to animal experimentation Legal status of embryos			25 min
Methods of gene transfer	Gene transfer through gametes, primordial germinal cells, somatic cells (i.e. nuclear cloning).			60 min
Theoretical models	Indirect strategies (siRNA, negative dominant, luring, gene fishing) and direct strategies (homologous recombination, positive and negative selection, gene reporting)			120 min
Application of transgenic animals	Transgenic animals as a source of biological material (myostatin knock‐out, muscle dystrophia, nuclear cloning in pigs, xenotransplantation, …) This section of the course is open to suggestions from students			120 min

The course stated that experimentation on animals must be performed in accordance with laws, acknowledging that laws are evolving under societal and scientific pressures. As a citizen, the student was invited to think over the multiple ethical issues raised by animal experimentation or genome editing.

### Protocol texts for surveys

Six protocols, A to F, were used in the survey. For third‐year bachelor students majoring in BCP, protocols A & B were used in October 2016, protocols C & D were used in December 2016, and protocols E & F were used in April 2017. Protocols A, C and D were used with third‐year bachelor students majoring in BOP.

#### Protocol A

‘The test described by Draize and Kelley is a standard approved procedure for testing eye irritation. It requires the use of animal models where tears are produced (lemur microcebus). In one eye of each of nine animals, 0.1 mL of the test product is instilled. In three animals, the product is then washed with 20 mL of warm water 2 s after instillation, and for three others, 4 s after instillation. In the last three, the product is left in contact with the eye. Ocular reactions are observed with the naked eye or with a luminous slot at 1, 2, 3, 4, and 7 days after treatment. The reactions of conjunctiva (redness, chemosis and secretion), cornea (degree and magnitude of opacity) and iris (congestion, swelling and injunctivity of the pericorneal region) are measured on a specific scale. Is the experimental protocol satisfactory?’ The text of the protocol is inspired by the test used by Draize and Kelley [26].

#### Protocol B

‘This describes a study of the Y gene, which is one of the early markers of cartilage differentiation. The two forms of this gene are expressed in distinct ways and can have opposite roles on the maturation of chondrocytes. To determine the role of this gene during cartilage formation, an overexpression of the Y gene is targeted through a specific promoter (promoter of the alpha 1 chain of type IV collagen). A mutated, negative dominant form is also over‐expressed in cartilaginous and bone tissue. Cellular lines of chondrocytes and osteocytes will be obtained after *in vivo* sampling at the limb bud or ribs after stage dissection at E11.5 (11.5 post‐fertilization days) and E18.5 (18.5 post‐fertilization days), respectively. These cell lines will be compared to the control cells, expressing the wild form of the Y gene and the inactive truncated form of the Y gene. Adult mouse phenotypes will also be analyzed [RT‐PCR, Hybridization *in situ* (HIS), macroscopic observations, clinical examinations, x‐rays]. Is the experimental protocol satisfactory? ’ The text of the protocol is inspired by Tsumaki et al. [27, 28] and Kozhemyakina *et al*. [27, 28].

#### Protocol C

‘Experimental models of chronic colitis to investigate the efficacy of treatments for chronic inflammatory bowel disease. Five groups of animals (*Rattus norvegicus*) are used for each pharmacological agent tested: Group 1 = control group without treatment, Group 2 = colitis induction with solvent treatment, Group 3–5 = Colitis induction with pharmacological agent treatment, respectively, at low, medium or high doses. The colitis induction protocol uses the solvent trinitrobenzene sulfonate (TNBS) at a dose of 150 mg·kg^−1^ for groups 2–5. Each experimental group consists of 16 animals. TNBS is administered intra‐rectally. The administration of anesthetics and analgesics/anti‐inflammatory drugs may decrease the effectiveness of the model and will therefore be excluded. The route of administration is intragastric, by gavage (10 mL·kg^−1^). Daily monitoring of animals is carried out. This protocol includes a total number of animals of 1440 for the test of ten pharmacological agents. A score of the severity of the pathology will be established daily for 1 week according to three criteria: the percentage of weight loss, the consistency of the faeces and the presence of blood in them. Inflammation of the intestine will be analyzed histologically. Is the experimental protocol satisfactory?’ The text of the protocol is inspired by Scheiffele *et al*. [29].

#### Protocol D

‘Hypoxia can cause severe fetal sequelae. Current methods for detecting acidosis and hypoxia are invasive (e.g. pH at scalp or lactate at scalp). The development of non‐invasive tools is necessary. To validate a new model for monitoring fetal suffering by analyzing the variability of the fetal heart rhythm, a protocol is proposed: implantation of a system for detecting cardiovascular parameters, prior to any subsequent experimentation. It includes 40 pregnant females, of the species *Sus scrofa domestica* (pig). After hysterectomy, vascular catheters (an arterial catheter and a venous catheter) and three subcutaneous electrodes, as well as a flow meter around the umbilical artery are inserted. A venous catheter is placed in the mother. Surgery is performed under general anesthesia (Sodium Thiopental, 1 g per 40 mL). Fetal analgesia during the operation is ensured by maternal anesthesia, and by intramuscular injection of buprenorphine (0.04–0.06 mL·kg^−1^) and subcutaneous 1 mL of lidocaine hydrochloride into the skin incision path. Antibiotics are administered intravenously (0.2 g trimethorprime, 1.2 g sulfadoxine). Losses of amniotic fluid during the operation are compensated by the supply of heated saline solution. Post‐operative analgesia is routinely performed. This protocol allows for subsequent, chronic, multi‐day experiments without further intervention on the mother or fetus. Is the experimental protocol satisfactory? ’ The text of the protocol is inspired by experiments performed in sheep [30, 31].

#### Protocol E

‘This protocol aims to analyze the function of lymphocytes in experimental models of multiple sclerosis, the pathological process of which is known as experimental autoimmune encephalomyelitis (EAE). Analysis of the severity of the disease is powerful in the EAE. Due to clinical scores ranging from 0 and 5, variations can be analyzed over time. Thus, the effects of the experimental conditions will be analyzed on the induction and chronicity of the disease. A ‘body condition score’ (BCS) is used to assess the progression of the disease in EAE rodents (0: No signs; 1: atonic tail; 2: paresis of the hind limbs; 3: paraplegia of the hind limbs; 4: quadri‐paresis; 5: quadriplegia/moribond). BCS will be monitored daily for 40 days. This protocol includes 288 rodents (mice).The EAE is induced in mice by immunization via a per cutaneous injection of a peptide emulsion (M, 100 μg) or human recombinant protein (M, 100 μg) (day 0) and two intravenous injections of pertussis toxin (100 μL, 200 ng) on the day of immunization. Debilitating signs of pathology occur after 10 days. Lymphocytic cells will be isolated from the spleen and lymph nodes 5 days after immunization. Specific lymphocytic populations will be isolated. The cellular responses will be characterized by proliferation and differentiation markers (Foxp3, IL‐17, IFNg, GMCSF, …), by flow cytometry and measurements of secreted cytokines. Is the experimental protocol satisfactory?’ The text of the protocol is inspired by models for EAE available in the literature [32, 33].

#### Protocol F

‘The test described by Draize and Kelley is a standard approved procedure for testing eye irritation. It requires the use of animal models where tears are produced (rabbits). In one eye of each animal, 0.1 mL of the test product is instilled; the other eye is a control. In one third of the animals, the product is then washed with 20 mL of warm water 2 s after instillation, and for another third, 4 s after instillation. For the last third of the remaining animals, the product is left in contact with the eye. Ocular reactions are observed with the naked eye or with a luminous slot at 1, 2, 3, 4, and 7 days after treatment. Reactions of conjunctiva (redness, edema and secretion), cornea (degree and magnitude of opacity) and iris (congestion, swelling and inflammation of the pericorneal region) are measured. The number of animals enrolled for each product has been reduced to 18 rabbits. Based on results *in vitro* studies, only two concentrations per product to be tested will be selected. Observation of any of several markers for potential suffering of animals during experimentation including weight loss > 20%, breathing abnormalities, behavior (aggressiveness, prostration, hyperactivity), or advanced dehydration, the experiment will result in the immediate cessation of an experiment and euthanasia of the animal. Is the experimental protocol satisfactory?’ The text of the protocol is inspired by the test used by Draize and Kelley [26].

## Results

### Students attitudes towards premises and moral dilemma during the lecture

#### Exposing the value of utilitarianism within the student classroom

The first interactive sequence of the course aimed to present current utilitarianism and evaluate its adoption by students. Regarding the proposal ‘It’s wrong to kill one of our own’, a large majority of students votes took the position that, indeed, it is wrong to kill one of our own. A majority (85.6 ± 3.6%) of students voted this proposal as true, whereas 14.4 ± 3.6% of students rejected the proposal (*n* = 191 students; three different lectures in 2017, 2018 and 2019).

On the other hand, a large majority of students voted that the death of a small number of people is acceptable in a critical situation, when facing a moral dilemma (at the helm; Table 1). Two options were proposed in that situation: (a) to turn off the oxygen and kill four sailors to prevent the spread of a fire and save the boat or (b) to not to turn off oxygen and have everyone die. A majority (80.7 ± 8.4%) of the students (*n* = 206 students; three different lectures in 2017, 2018 and 2019) voted to turn off the oxygen and thereby validate the sacrifice of a small number for the greatest happiness or security of the greatest number. The adoption of the values of utilitarianism by a majority of students was briefly debated during the lecture.

#### Are animals or embryos one among us?

The second interactive sequence of the course aimed to make students aware of the definition of us. The first proposal was that ‘animals are one of us’ and the second one was that ‘embryos are one of us’. We observed that students tend to consider animals to be among their own; 72.6 ± 6.6% of the students believed the proposal to be true, whereas 27.4 ± 6.6% of students considered that animals are not one among us (*n* = 202 students; three different lectures in 2017, 2018 and 2019). Nevertheless, the results of the second proposal showed that embryos are not immediately recognized as being part of us. Indeed, the proposal was estimated to be true for 44.8 ± 11.2% of the students (*n* = 200 students; three different lectures in 2017, 2018 and 2019), whereas 55.2 ± 11.2% of the students did not consider embryos as one among their own.

### Protocol survey: effects of the course on third‐year undergraduate students majoring in BCP

#### Before lectures

The survey was performed before the course, in October 2016. Students were asked to examine protocols A and B.

Protocol A proposed in this survey presented an animal experiment (eye irritation) on a species (lemur) that is protected by the Convention on International Trade in Endangered Species of Wild Fauna and Flora (CITES), also known as the Washington Convention. Expected responses included a reminder of protected species legislation, an empathic response (consideration of animal suffering), and even the refusal of any animal experimentation. Responses were discriminated as: (a) replies referring to the term ethics or a situation with an ethical dimension; (b) yes/no replies without justification; and (c) replies with arguments other than referring to ethics. Analysis of the responses showed that 30 of 137 students (21.9%) cited the issue of ethics in their rationale for whether or not to accept this experimental protocol (Table 2). Among them, six accepted the protocol without sharing a supportive argument, although they mentioned the term ‘ethics’ in their response. Three students did not provide a definitive answer but nevertheless constructed an argument around animal suffering. Almost one‐half of the students rejected it without supportive arguments. Finally, among the eleven students who rejected the protocol based on a supportive argument, eight evoked animal suffering and three relied on legislative references. We therefore noted that, at the end of this first protocol, few students showed spontaneously sensitive behavior to the ethical questions of animal experimentation.

**Table 2:** Analysis of students’ response to protocols A and B (third‐year undergraduate students mentioning in Cellular Biology and Physiology).

	Yes, the protocol is not satisfactory	No, the protocol is not satisfactory	Not clear‐cut opinion	Total
Protocol A				
Students mentioning an ethical dimension	6	21	3	30 (21.9%)
Supported by a legislative argument	0	3	0	3
Supported by a argument on animal suffering	0	8	3	11
No argument	6	10	0	16
Students discussing other dimensions than ethical ones	38	34	13	85 (62. 1%)
No discussion	12	5	5	22 (16.0%)
Total	56 (40.9%)	60 (43.8%)	21 (15.3%)	137
Protocol B				
Students mentioning an ethical dimension	1	5	3	9 (6.6%)
Supported by a legislative argument	0	0	0	0
Supported by a argument on animal suffering	0	0	0	0
No argument	1	5	3	9
Students discussing other dimensions than ethical ones	21	20	26	67 (48.9%)
No discussion	41	5	15	61 (44.5%)
Total	63 (45.9%)	330 (21.9%)	44 (32.2%)	137

The second proposed protocol (B) of the survey presented an animal experiment (animal transgenesis) including vivisection of mammalian embryo (mouse) limb buds. Expected responses included a reaction to vivisection, an empathic reaction (consideration of animal suffering) and even the refusal to condone any animal experimentation. Only nine of 137 students (6.6%) responded to protocol B by questioning the need for vivisection, and none relied on a built case. Here, the students appeared to completely fail to detect and explain any potential ethical problems raised by this protocol (Table 2).

#### Short‐term impact of the course

A second survey was performed in December 2016, at the end of the winter semester. Students were asked to examine protocols C and D.

The third protocol (C) presented an experimental device (pharmacological agent test) of an induced colitis model. Expected responses included a reaction on the absence of anesthesia and analgesia displayed in the protocol, a reaction on the number of animals used (3Rs: reduce; animal number enrolled was overestimated), an empathic reaction (consideration of animal suffering, gavage) and even the rejection of any animal experimentation. Almost three‐quarters of students (70%) responded to the protocol by raising an ethical issue (Table 3). Twenty‐six 89 students (29.2%) talked about a legislative argument or the ‘3Rs’ rule and animal suffering. Twenty‐two of 89 students (24.7%) pointed out the legislative argument and the ‘3Rs’ rule. Fourteen 89 students (15.7%) only referred to animal suffering. We noted that all respondents provided supportive arguments for their ethical position this time.

**Table 3:** Analysis of students’ response to protocols C and D (third‐year undergraduate students mentioning in Cellular Biology and Physiology).

	Yes, the protocol is not satisfactory	No, the protocol is not satisfactory	Not clear‐cut opinion	Total
Protocol C				
Students mentioning an ethical dimension	7	37	18	62 (70%)
Supported by a legislative/3Rs argument	3	13	6	22
Supported by a argument on animal suffering	2	9	3	14
Supported by arguments on animal suffering and on legislation/3Rs	2	15	9	26
No argument	0	0	0	0
Students discussing other dimensions than ethical ones	8	3	10	21 (23.6%)
No discussion	4	2	0	6 (7%)
Total	19 (21.3%)	42 (47%)	28 (31.5%)	89
Protocol D				
Students mentioning an ethical dimension	15	13	20	48 (54%)
Supported by a legislative/3Rs argument	0	0	0	0
Supported by a argument on animal suffering	13	12	17	42
Supported by arguments on animal suffering and on legislation/3Rs	2	1	3	6
No argument	0	0	0	0
Students discussing other dimensions than ethical ones	4	1	12	17 (19%)
No discussion	17	1	6	24 (27%)
Total	36 (44.4%)	15 (16.9%)	38 (42.7%)	89

The fourth protocol (D, the second of this survey at this time) described the implantation *in utero* of a system for detecting cardiovascular parameters. Expected responses included a reaction on the manipulation of the embryo, an empathic reaction (consideration of animal suffering) or even the rejection of any animal experimentation. One‐half of the students [48/89 (54%)] (Table 3) mentioned ethics in their comments. All were based on an argument related to animal suffering. A few [6/89 students (6.7%)] also mention the 3Rs and/or a legislative argument. Several students wrote that they did not understood the protocol because they failed to understand the scientific vocabulary used.

#### Long‐term impact of the course

Finally, students were asked to examine protocols E and F at the end of the academic year, in April 2017, more than 6 months after the intervention.

Protocol E described an animal experiment including a functional analysis of a cell population in mouse models of multiple sclerosis (EAE). Expected responses included a reaction to the absence of analgesia proposed in a clearly painful protocol, a reaction to the number of animals used (lack of justification for the number of animals used, 3R), an empathic reaction (consideration of animal suffering induced by the EAE protocol) and even the rejection of any animal experimentation.

Protocol F was an amendment of protocol A (eye irritation). The protocol proposed in this assessment no longer presented an animal experiment on a protected species by the CITES (Washington Convention), but instead one on the rabbit, which is protected by European directives. The protocol was amended to clarify the consideration of animal suffering (limit points) and to include a justification of the number of animals used. Expected responses included a discussion of the ethical nature of the protocol (3R), an empathic reaction and even the rejection of any animal experimentation.

**Fig. 2:**
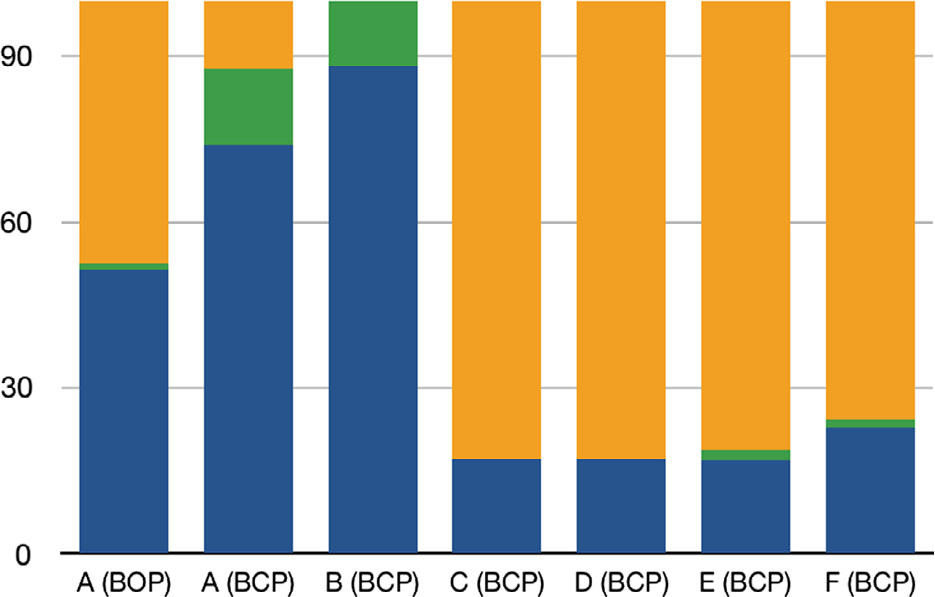
Histogram of student responses regarding the development of ethical dimensions in the critical comments of the protocols (normalized, with 100% representing total critical comments written by students). Details are provided in Tables 2, 3, 4 and 6. 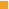 Students discussing an ethical dimension in their responses. 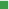 Students writing ‘ethical’ or ‘ethics’ in their responses, without discussing it. 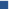 Students providing other critical comments than ethics to the protocol. BOP, Biology of Organisms and Populations; BCP, Cellular Biology and Physiology.

For protocol E, the majority of students raising an ethical concern [48/49 (97.6%)] justified it and/or made a brief argument relying on the 3Rs principle (20/49) (Table 4). For protocol F, students raising an ethical concern [50/54 (92.6%)] justified it and/or made a brief argument relying on the 3Rs principle (44/54) (Table 4). For analysis, the results were normalized to the total number of critical comments written by the students (respectively, 115, 76, 83, 65, 59 and 70 for A, B, C, D, E, and F). Histograms are provided in Fig. 2.

**Table 4:** Analysis of students’ response to protocols E and F (third‐year undergraduate students mentioning in Cellular Biology and Physiology).

	Yes, the protocol is not satisfactory	No, the protocol is not satisfactory	Not clear‐cut opinion	Total
Protocol E				
Students mentioning an ethical dimension	17	18	14	49 (37.4%)
Supported by a legislative/3Rs argument	2	4	7	13
Supported by a argument on animal suffering	12	11	5	28
Supported by arguments on animal suffering and on legislation/3Rs	3	2	2	7
No argument	0	1	0	1
Students discussing other dimensions than ethical ones	2	0	8	10
No discussion	37	13	22	72
Total	56	31	44	131
Protocol F				
Students mentioning an ethical dimension	19	10	25	54 (41.2%)
Supported by a legislative/3Rs argument	12	3	15	30
Supported by a argument on animal suffering	3	2	1	6
Supported by arguments on animal suffering and on legislation/3Rs	3	4	7	14
No argument	1	1	2	4
Students discussing other dimensions than ethical ones	5	0	11	16
No discussion	29	11	21	61
Total	53	21	57	131

### Protocol survey (third‐year undergraduate students majoring in BOP)

Although the students involved in the BCP program appeared to be less sensitive to the ethical aspects of animal experimentation (Table 2), we considered whether students in a field where the animal is at the center of the teachings are more likely to distinguish an ethical concern in an experiment. In such a case, their values and attitudes related to animal experimentation should exhibit differences. We therefore submitted three protocols (A, C and D) for evaluation by the third‐year students majoring in BOP, in December 2016. It should be noted that the two cohorts of students were enrolled in completely independent courses, and that students majoring in BOP did not take any courses in ethics or bioethics. Among those students, 40 of 79 (50.6%) students identified an ethical concern in protocol A (Table 5). Similar results were obtained for protocols C and D because 44 of 79 (55.7%) and 38 of 79 (48.1%) students, respectively, mentioned ethical concerns (Table 5). These results are higher than those of undergraduates majoring in BCP, among whom only 30 of 137 (21.9%) noted ethical concerns in protocol A, compared to 40/79 (50.6%) of students who majored in BOP (Table 6).

**Table 5:** Percentage of students mentioning an ethical dimension when evaluating the protocols. BOP: mentioned by third‐year undergraduate students in Biology of Organisms and Populations, *n* = 79 students; BCP: mentioned by third‐year undergraduate students in Cellular Biology and Physiology, *n* = 137 students for protocols A and B, *n* = 89 students for protocols C and D, *n* = 131 students for protocols E and F. ND, no determined.

Protocol	A	B	C	D	E	F
BCP: Students mentioning an ethical dimension	22%	7%	70%	54%	37%	41%
BOP: Students mentioning an ethical dimension	51%	ND	56%	48%	ND	ND

**Table 6:** Analysis of students’ response to protocols A, C and D (third‐year undergraduate students majoring in Biology of Organisms and Populations).

	Yes, the protocol is not satisfactory	No, the protocol is not satisfactory	Not clear‐cut opinion	Total
Protocol A				
Students mentioning an ethical dimension	2	11	27	40 (5.6%)
Supported by a legislative/3Rs argument	1	2	4	7
Supported by argument on animal suffering	1	6	22	29
Supported by arguments on animal suffering and on legislation/3Rs	0	3	0	3
No argument	0	0	1	1
Students discussing other dimensions than ethical ones	4	1	31	36 (45.6%)
No discussion	1	0	2	3 (3.8%)
Total	7	12	60	79
Protocol C				
Students mentioning an ethical dimension	4	6	34	44 (55.7%)
Supported by a legislative/3Rs argument	1	0	10	11
Supported by argument on animal suffering	2	4	14	20
Supported by arguments on animal suffering and on legislation/3Rs	1	1	7	9
No argument	0	1	3	4
Students discussing other dimensions than ethical ones	7	1	15	23 (29.1%)
No discussion	6	1	5	12 (15.2%)
Total	17	7	54	79
Protocol D				
Students mentioning an ethical dimension	10	2	26	38 (48.1%)
Supported by a legislative/3Rs argument	0	0	1	1
Supported by argument on animal suffering	9	2	24	35
Supported by arguments on animal suffering and on legislation/3Rs	0	0	0	0
No argument	1	0	1	2
Students discussing other dimensions than ethical ones	7	0	11	18 (22.8%)
No discussion	13	0	10	23 (29.1%)
Total	30	2	47	79

For analysis, the results were normalized to the total number of critical comments written by the students (76 students out of 79) and compared with those of the students majoring in BCP (Fig. 2).

### Online attendance for critical examination of animal experimentation protocols made available to students

From October 2017 up to April 2019, the six protocols used for the survey were proposed online to the 320 students who were registered on the courses. Using the online application socrative (http://socrative.com), students were able to read and anonymously provide their criticisms and remarks about the protocols. After each response, the student was able to access a short text summarizing the expected reactions and potential ethical issues raised by the protocols. Of the 320 students registered in these courses, 60 students (18.75%) examined the first protocol, whereas only 38 students (11.88%) read and examined all six protocols.

## Discussion

The present survery was performed aiming to evaluate, in large classrooms, students' opinions on ethical issues, as well as to understand the ability of students to exercise critical thought on experimental protocols that include animal experimentation. We also wanted to evaluate the impact of teaching in large, interactive groups. We chose to use the context of a lecture with respect to the use of transgenic animals in science because the latter topic is at the crossroads of ethical issues concerning animal experimentation, embryo manipulation, genome editing and human/non‐human chimeras. A lecture scenario was built (Table 1) based on utilitarian valuation, critical thinking and interactivity. The first hour of the course was designed to present, on the one hand, the process of evaluation (i.e. to determine what we hold as valuable) and, on the other hand, the standards of duty and obligation applied in the field (deontology). Such a course was therefore designed to create a learning environment for increasing the motivation, ethical awareness and participation of the students. Besides focusing on the student, another goal of the present study was to underline the implicit values conveyed consciously or not, in a lecture. Data and results are presented as objective, or are presented as data submitted to a reviewer's critical exercise. It is difficult for students to question these observations; in addition to raising doubts about the objective nature of the data itself, criticisms also question the credibility of both the people who evaluated the work, as well as the people presenting the body of work, including the lecturer. Raw data may contain some subjectivity, depending on how they are obtained (e.g. in biology and cellular imaging) [34]. The data from this survey were analyzed for the ability of students to express a critical analysis of the ethical aspects of animal testing protocols. The intrinsic nature of students' responses to the adequacy of protocols with regard to ethical rules is beyond the scope of the present study.

Thus, the aim of the lectures was to promote awareness of animal welfare through both an understanding of the process of the scientific works and their outcomes, and by developing an implicit value inherent to teaching animal transgenesis. Why? First, it is necessary for students to be able to realize that animal experiments, as well as transgenic experiments, take place within a set framework and limits. Second, animal experimentation is the object of a moral experience and a valuation process. It can create conflicts between values that should not be concealed. Failure to consider and address any conflicts between values that may be raised by animal experimentation prevents a moral representation of these animal experiments. Universities have a moral duty to consider these potential conflicts and to hold values of ethics in this context. The core of the debate on animal experimentation relies on the moral question of the relationships between human and non‐human animals. The notion that human beings are superior to non‐human animals has become less tenable [35]. The book ‘’animal liberation’ [36] brought the debate on animal suffering, including that justified in the name of science, to a very large audience. According to the consequentialist or utilitarian position that originated in Europe in the late 18th and early 19th Centuries, animal research appears justifiable when it provides a substantial benefit for humans. Indeed, for theorists J. Bentham and J. S. Smith, the right acts shall produce the greatest amount of good consequences for the greatest number of beings. Therefore, a cure for an incurable disease or condition might justify the experiments. On the other hand, if animals are considered to have rights themselves [37, 38], there is no justification to exploit them for experimentation, irrespective of any possible benefit. Although these debates and positions are more and more commonly exposed in society, a few students perceived that the introduction of these debates into the classroom was intrusive because they felt that ethics or philosophy should have no place in a course on molecular or cellular biology.

The first interactive sequence of the lecture aimed at evaluating the acceptance of the utilitarian position. We observed the majority of the students adopted the value that ‘it is wrong to kill one of us’ (85.6%; *n* = 191 students), as well as the death of a small group among us is acceptable when facing a moral dilemma (80.7%; *n* = 206 students). Therefore, utilitarianism did not raise critical issues for the students in the classroom. Nevertheless, caution is warranted when evaluating moral dilemmas. First, cultural factors must be taken into account when addressing a sacrificial dilemma [39]. When a similar sequence of lectures was used with Chinese students, the description of a sacrificial dilemma initiated debates between students and with the lecturer, and raised, in an acute manner, the question of the right of life or death on others with these students. It is notable that the Chinese students were explicitly exposed during their studies to the consequentialist philosophy of Mozi [40] and to have a more acute perception of utilitarianism (J.F. Bodart, personal observations). Second, this type of problem may be used as an ‘intuition pump’ to help an audience understand the notion of utilitarianism. As an experimental paradigm, it has its own limits. Many sacrificial dilemmas may appear absurd, artificial and frivolous, and also appear to differ fundamentally from more realistic dilemmas. It should also be considered that such scenarios are far too extreme and unconnected to real‐life moral situation to be useful or educational [39, 41]. These concerns must be explained to students so that they can undertake a more reflexive and critical attitude toward the valuation process during the course.

A second dimension of the interactive sequence was to raise the awareness of our ability to acknowledge non‐human animal beings or human embryos as ‘one of us’. Some 72.6 ± 6.6% of the students believed the premise ‘the animals are one of us’ to be true, and therefore acknowledged the values associated with animal welfare when non‐human animals are considered to be sentient beings. To the question ‘are embryos one among us?’, the reaction of the students was less clear, and they considered the embryo not to be one of them (55.2%; *n* = 200). During discussions in the large classrooms, students acknowledged that their responses were conditioned by their representation of the embryo, either thought of by students as a fetus or by other students as early embryos being equal to a bundle of cells. Thus, the importance of the representation was clearly institutionalized during the lecture, to make more explicit the valuation process involved in the ability to accept non‐human animals or embryos as ‘one of us’.

Students tend to consider the animal as a scientific object. Indeed, few of the BCP students commented on the ethical problems in protocols A and B before the course. Students who note ethical problems mostly reject the protocols. Thus, before the course, students mainly consider the animal as an object in a scientific context. This representation of the animal is in contrast to the statement that the animal is one of ours.

On the other hand, students in BOP show another representation of the animal related to the implicit values of the lessons that they follow; namely, that the animal is considered as a sentient subject. In this student population, half of the students report on ethical issues in the protocols (protocols A, C and D) (Fig. 2 and Table 3). Then, depending on the students, the implicit values or representations regarding animal manipulation are different. Students in a field where the animal is at the center of the teachings are more likely to distinguish an ethical dimension in an animal experimentation protocol and to take the animal out of a solely scientific perception. It should be noted that the two cohorts of students follow completely independent courses, and that no courses on ethics or animal experimentation are provided to the students majoring in BOP. Because none of the students of the BOP cohort mentioned the principle of the 3Rs, there is no reason to believe that the BCP students discussed the material with their BOP peers. Assuming that this is the case, their values and attitudes related to animal experimentation are indeed different. The attitude of these students did not rely on the knowledge of the 3Rs or laws, but instead on empathy, by mainly considering the suffering of the animals. By contrast, students in BCP find it difficult to spontaneously remove the animal from its scientific significance and consider it as part of an experimental protocol. They therefore have difficulties with respect to conciliating the values they acknowledged for animal welfare and the implicit values anchored in utilitarianism within the transgenesis approaches.

The course appeared to have a short‐term effect on students’ views of the animal as an object versus a sentient being; after the course, 54–70% of students have an ethical concern compared to 7–22% before the course (Tables 4 and 5). What remains of this ability several months after the course? On analysis of protocols E and F, 37–41% of students (i.e. two out of five students) remain aware of ethical issues. If we look at the number of students giving a critical opinion on the protocols before the course, just after the course and several months after the course, there are no dramatic fluctuations (Table 7). If we consider the nature of the critical discussion of the protocol, it evolves to be primarily ethical after the course, and remains primarily oriented to ethics several months after the course. Because the number of students critically discussing the ethical points of the protocols increased significantly, it is tempting to consider the hypothesis that, among those students who carry out a critical analysis and verbalize it, there was an anchoring of ethical issues because ethical and legislative arguments were strongly expressed (Tables 3 and 4). We cannot discard the hypothesis that students rejected or accepted the protocol without expressing the ethical problems that the reading of the protocols may have raised. Such students integrated the critical process of ethical dimensions without verbalizing it.

**Table 7:** Undergraduate students (majoring in Cell Biology end Physiology) mentioning an ethical dimension when evaluating the protocols, relatively to the students discussing the protocols.

Protocol	A	B	C	D	E	F
*n* Students providing critical comments to the prococol/*n*’ total students	115/137	76/137	83/89	65/89	59/131	70/131
Students mentioning ethical dimension in the developed responses	30	9	62	48	49	54
Students discussing ethical dimension in the developed responses	14	0	62	48	48	53

The understanding of an ethical decision in science relies on the understanding of both the process of the scientific work and its outcomes. Students need a grasp of the basic biology before they can make an intelligent ethical decision or statement on a new technology. Ignorance may fuel controversy about what can or cannot be done [34]. Therefore, whether the students consider themselves sufficiently informed and believe that they have a sufficient scientific background to understand the methodologies and the protocols might be questioned. According to a survey performed on the same student population, a majority of the students consider themselves to have a sufficient scientific background to understand the proposed protocols (data not shown).

A difference was observed between the proportion of students identifying ethical issues on protocols B and A. This difference might be a result of protocols themselves because the protocols are not strictly identical and one protocol was more difficult to understand than the other. It might also correlate with a lack of time; students may have spent a considerable time on the first scenario and therefore had less time to analyze the second scenario. In this case, the students would give an unsupported opinion on the second protocol and a more structured opinion on the first protocol. This hypothesis appears to be supported by the fact that, between scenarios A and B, as well as between scenarios C and D for the cohorts of BCP students, there was an increase in the number of unsupported responses [opinion without arguments: A, 22/137 (16%); B, 61/137 (44.5%); C, 6/89 (7%); D, 24/89 (27%). A similar dynamic was observed in the cohort of BOP students where responses without supportive arguments was different in each scenarios (A, 3/79 (3.8%); C, 12/79 (15.2%) and D (29.1%)]. However, such a difference was not observed between scenarios E and F (‘no discussion’ group included 72/131 students for protocol E and 61/131 for protocol F).

Could online exercises about critical examination of animal experimentation help to reinforce the ethical points raised in a course? The answer is rather negative because, in our case, students showed a very low interest in such a proposal. When animal experimentation protocols were provided online to students in 2017, 2018 and 2019, in parallel to the course, only 11.88% of students (*n* = 320) read and examined each of the six protocols. This observation suggests that there may be a better uptake for students to address ethics in groups, rather than individually in front of a computer. Nevertheless, other reasons for the poor uptake of the online exercises may be that the exercises are voluntary or that the students have a high workload and did not have time to complete the exercises.

Large classrooms are not considered to be relevant places for questioning ethical issues because both the intensity and quantity of interactions between teachers and student are reduced as the classroom size increases, and this may promote passivity among students [12, 13]. However, in our hands, questions raised by the interactive sequences of the course highly motivated the students to participate and express their opinions, and, for some of them, to address their perceptions of living beings. We have observed that (a) a large number students of BCP had a fixed perception of the animal as a scientific object and struggled to take the animal out of its scientific context and consider it as a sentient being and (b) students showed a great interest in interactive sequences through excellent participation rates. The 1‐h course had a short‐term effect on students, changing their perception of the animal in a scientific context and increasing their sensitivity to ethical questions. We observed that this effect persisted in a modest way in the long term because the proportion of students discussing ethical aspects of protocols increased shortly after the lecture and remained elevated at the end of the academic year. When comparing the number of students explicitly discussing an ethical concern (Table 7) between the initial situation (protocol A) and shortly after the course (C and D), there was an increase from 3.4‐ to 4.4‐fold. When comparing the number of students explicitly discussing an ethical concern (Table 7) between the initial situation (protocol A) and shortly after the course (E and F), there was an increase from 3.4‐ to 3.8‐fold. The surveys were collected anonymously and did not allow us to track individual students over the academic year, and so there is no way to confirm that students explicitly discussing ethical concerns were the same on the second and the third occasions. These observations nevertheless suggest that the effect of teaching has not faded or dissipated, even though the lecture was performed in a large classroom. These observations fueled our motivation to adopt a reflexive position in a large classroom, and to promote interactivity in this context.

## Conflict of interests

The authors declare that they have no conflicts of interest.

## Author contributions

JFB and AD conceived and designed the project. JFB and AD acquired the data. JFB analyzed and interpreted the data. JFB and AD wrote the article.
